# Supplementary material for: Phylogenomic relationship and evolutionary insights of sweet potato viruses from the western highlands of Kenya
Source: PeerJ. 2018 Jul 19;6:e5254. doi: 10.7717/peerj.5254 (PMC6054865; doi:10.7717/peerj.5254)
Supplement: Supplemental Information 1 [file peerj-06-5254-s001.docx]

| Sample GenBank accession numbers | Virus | Geographical location |
| --- | --- | --- |
| SRF109a | Sweet potato virus C | Kenya |
| NC_014742 | Sweet potato virus C | Peru |
| GU207957 | Sweet potato virus C | Peru |
| KU511269 | Sweet potato virus C | Spain |
| KU877879 | Sweet potato virus C | China |
| JX489166 | Sweet potato virus C | Israel |
| KP115622 | Sweet potato virus C | South Korea |
| KP115620 | Sweet potato virus C | South Korea |
| KP115621 | Sweet potato virus C | South Korea |
| MF572061 | Sweet potato virus C | Australia |
| MF572057 | Sweet potato virus C | Australia |
| MF572058 | Sweet potato virus C | Australia |
| MF572059 | Sweet potato virus C | Australia |
| MF572067 | Sweet potato virus C | East Timor |
| KP115621 | Sweet potato virus C | South Korea |
| MF572064 | Sweet potato virus C | East Timor |
| MF572065 | Sweet potato virus C | East Timor |
| MF572066 | Sweet potato virus C | East Timor |
| MF572063 | Sweet potato virus C | Australia |
| MF572062 | Sweet potato virus C | Australia |
| MF572060 | Sweet potato virus C | Australia |
| NC_001841 | Sweet potato feathery mottle virus | Lab Isolates |
| D86371 | Sweet potato feathery mottle virus | Lab Isolates |
| KP115608 | Sweet potato feathery mottle virus | South Korea |
| KP115610 | Sweet potato feathery mottle virus | South Korea |
| KU511268 | Sweet potato feathery mottle virus | Spain |
| AB509454 | Sweet potato feathery mottle virus | Lab Cultures |
| AB465608 | Sweet potato feathery mottle virus | South Korea |
| KP115609 | Sweet potato feathery mottle virus | South Korea |
| AB439206 | Sweet potato feathery mottle virus | Lab cultures |
| SRF109a | Sweet potato feathery mottle virus | Kenya |
| FJ155666 | Sweet potato feathery mottle virus | Peru |
| MF572056 | Sweet potato feathery mottle virus | East Timor |
| MF572050 | Sweet potato feathery mottle virus | Australia |
| MF572053 | Sweet potato feathery mottle virus | East Timor |
| FJ155666 | Sweet potato feathery mottle virus | EastTimor |
| MF572055 | Sweet potato feathery mottle virus | EastTimor |
| MF572046 | Sweet potato feathery mottle virus | Australia |
| MF572049 | Sweet potato feathery mottle virus | Australia |
| MF572048 | Sweet potato feathery mottle virus | Australia |
| MF572047 | Sweet potato feathery mottle virus | Australia |
| MF572052 | Sweet potato feathery mottle virus | Australia |
| MF572051 | Sweet potato feathery mottle virus | Australia |
| MF572054 | Sweet potato feathery mottle virus | Australia |
| AJ428554 | Sweet potato chlorotic stunt virus | Uganda |
| NC_004123 | Sweet potato chlorotic stunt virus | Uganda |
| KC146842 | Sweet potato chlorotic stunt virus | China |
| SRF109a | Sweet potato chlorotic stunt virus | Kenya |
| KC888966 | Sweet potato chlorotic stunt virus | China |
| KC146840 | Sweet potato chlorotic stunt virus | China |
| KC888965 | Sweet potato chlorotic stunt virus | China |
| KC888964 | Sweet potato chlorotic stunt virus | China |
| SRF109a | Sweet potato chlorotic stunt virus | Kenya |
| NC_006550.1 | Sweet potato chlorotic fleck virus | Uganda |
| AY461421 | Sweet potato chlorotic fleck virus | Uganda |
| KP115607 | Sweet potato chlorotic fleck virus | South Korea |
| KP115606 | Sweet potato chlorotic fleck virus | South Korea |
| KP115605 | Sweet potato chlorotic fleck virus | South Korea |
| KU707475 | Sweet potato chlorotic fleck virus | Australia |
| KU720565 | Sweet potato chlorotic fleck virus | East Timor |
| KP715159 | Sweet potato chlorotic fleck virus | South Korea |
