# Supplementary material for: Phylogenomic relationship and evolutionary insights of sweet potato viruses from the western highlands of Kenya
Source: PeerJ. 2018 Jul 19;6:e5254. doi: 10.7717/peerj.5254 (PMC6054865; doi:10.7717/peerj.5254)
Supplement: Supplemental Information 2 — Only clades with posterior probability of above 0.7 were considered. [file peerj-06-5254-s002.docx]

| Virus | Clade I | P1 | P3 | Nlb | CI | Coat protein | 6K2 | 6K1 | Nla-Pro | Nla-vpg | HC-Pro |
| --- | --- | --- | --- | --- | --- | --- | --- | --- | --- | --- | --- |
| SPVC | Clade I | 0.53 | 0.81 | 1 | 0.98 | 1 | 0.94 | 0.94 | 1 | 0.96 | 0.84 |
|  | Clade II | 1 | 0.81 | 0.98 | 0.94 | 0.94 | 1 | 0.83 | 0.98 | 0.79 | 0.99 |
|  | Clade III | - | 0.78 | 0.99 | 0.97 | - | - | 0.79 | 0.93 | - | 0.92 |
|  | Clade IV | - | - | - | 0.56 | - | - | 0.69 | 0.75 | - | - |
| SPFMV | Clade I | 0.78 | 0.53 | 0.98 | 0.98 | 1 | 1 | 1 | 0.80 | 0.85 | 1 |
|  | Clade II | 1 | 0.71 | 0.99 | 0.89 | 0.97 | 0.83 | - | 0.70 | 0.78 | 0.99 |
|  | Clade III | 1 | - | 0.95 |  | 0.99 | 0.81 | 0.80 | 0.96 | 0.75 | 0.98 |
|  | Clade IV | - | - | - |  | - | - | 0.80 | - | - | - |
|  | Clade V | - | - | - |  | 0.98 | - | - | - | - | - |
