# Supplementary material for: Phylogenomic relationship and evolutionary insights of sweet potato viruses from the western highlands of Kenya
Source: PeerJ. 2018 Jul 19;6:e5254. doi: 10.7717/peerj.5254 (PMC6054865; doi:10.7717/peerj.5254)
Supplement: Supplemental Information 3 — Only clades with posterior probability of above 0.7 were considered. [file peerj-06-5254-s003.docx]

| Virus | Clade I | Triple block 1 | Triple block 2 | Triple block 3 | RdRP | Coat protein | Nucleic acid binding |  |  |  |
| --- | --- | --- | --- | --- | --- | --- | --- | --- | --- | --- |
| SPCFV | Clade I | 0.92 | 0.95 | 0.89 | 0.99 | 1 | 0.89 |  |  |  |
|  | Clade II | 0.99 | 0.90 | 0.72 | - | 0.72 | 0.79 |  |  |  |
|  | Clade III | 0.83 | 0.65 | 0.62 | 0.88 | 0.98 | - |  |  |  |
|  |  |  |  |  |  |  |  |  |  |  |
|  | **Clades** | **HSP70** | **Coat**  **Protein** | **mcp** | **P28** | **P60** | **P7** | **P8** | **RdRP** | **Rnase3** |
| SPCSV (RNA1&RNA2) | Clade I | - | 0.99 | 0.99 | 1 | 0.7769 | 0.62 | 1 | 0.99 | 1 |
|  | Clade II | 0.99 | 0.77 | 0.85 | 0.77 | - | 1 | 1 | - | 0.96 |
|  | Clade III | 0.81 |  | - | - | 1 | - | - | 0.98 | 0.78 |
|  | Clade IV | - |  |  | - | - | - | - | 0.99 | - |
