# Supplementary figures and images for: Phylogenomic relationship and evolutionary insights of sweet potato viruses from the western highlands of Kenya
Source: PeerJ. 2018 Jul 19;6:e5254. doi: 10.7717/peerj.5254 (PMC6054865; doi:10.7717/peerj.5254)

SVDQuartets bootstrap consensus

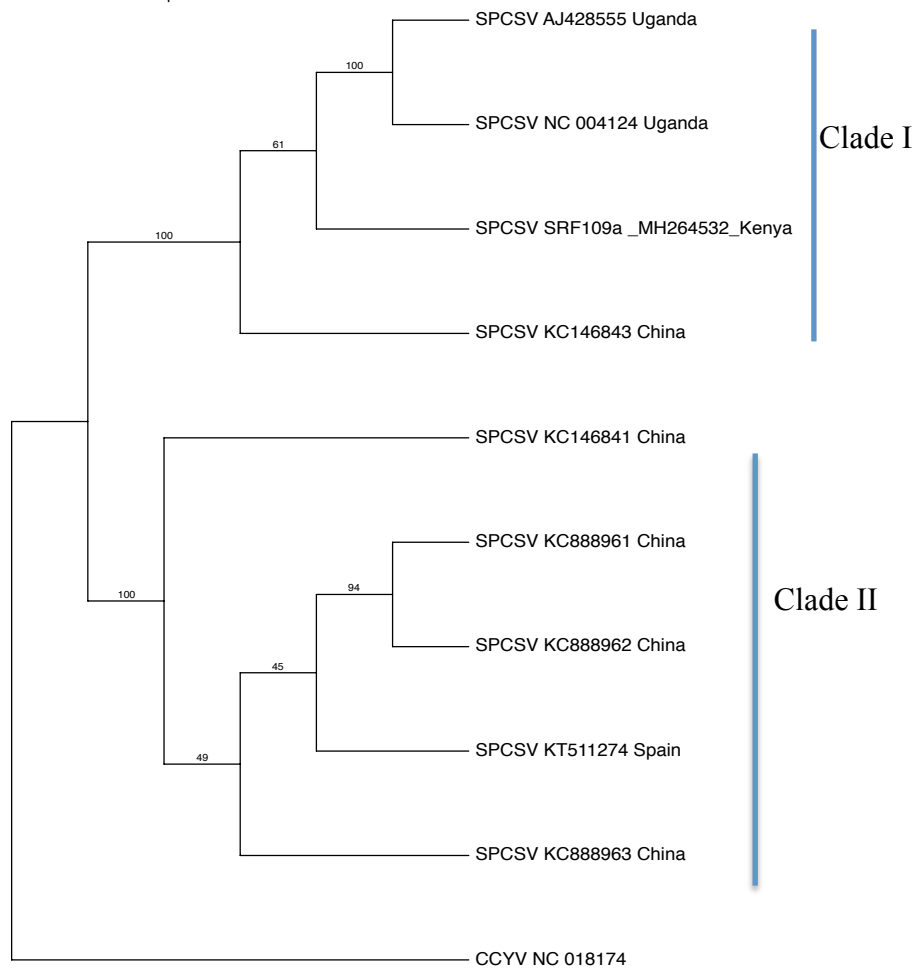

Supplement: Supplemental Information 4 — The nodes across each branch are labeled bootstrap values. Tip labels contain information of: virus name, GenBank accession number and/or field identification and country sampled. [file peerj-06-5254-s004.pdf]

SVDQuartets bootstrap consensus

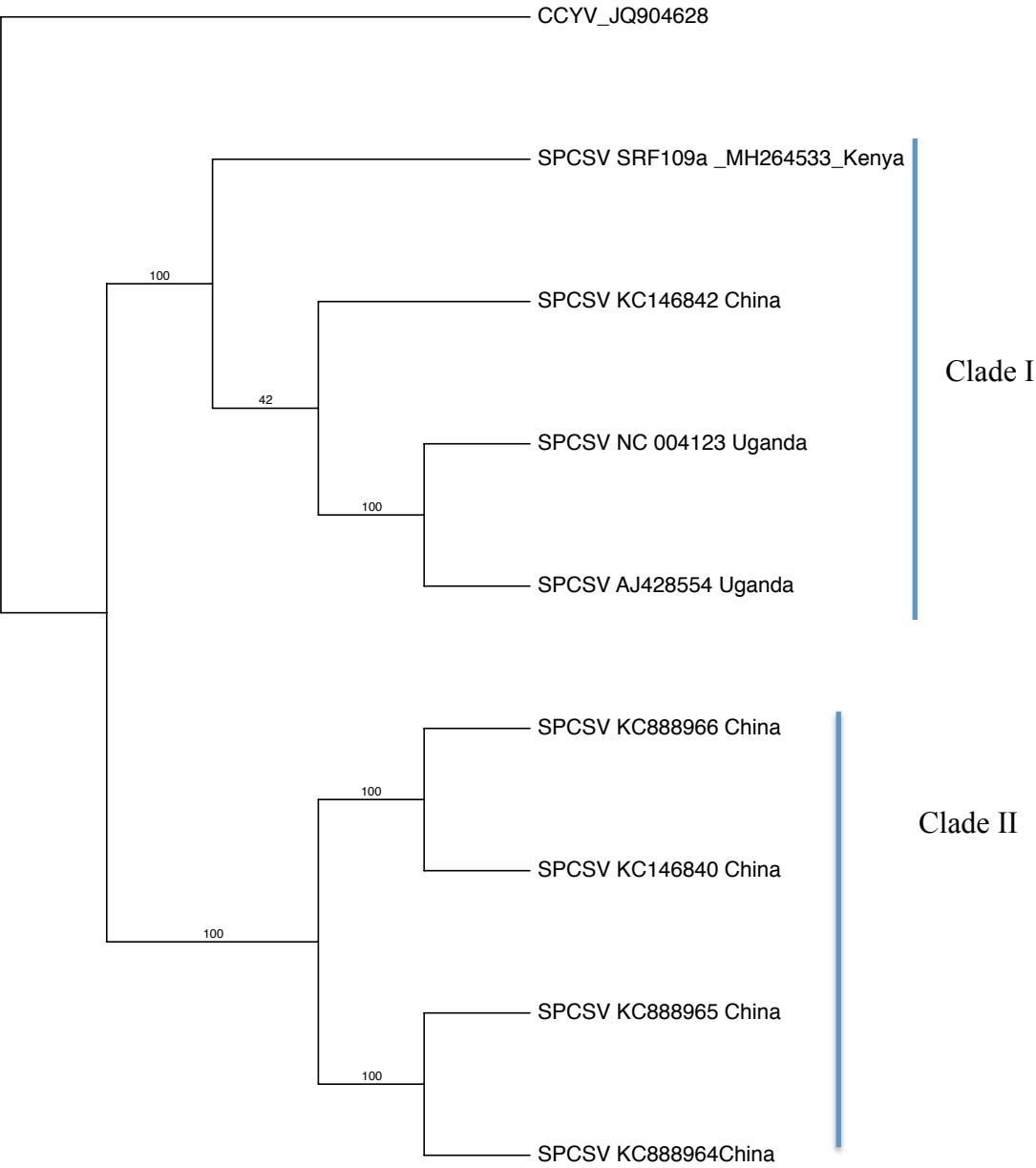

Supplement: Supplemental Information 5 — The nodes across each branch are labelled with bootstrap values. Tip labels contain information of: virus name, GenBank accession number and/or field identification and country sampled. [file peerj-06-5254-s005.pdf]

SVDQuartets bootstrap consensus

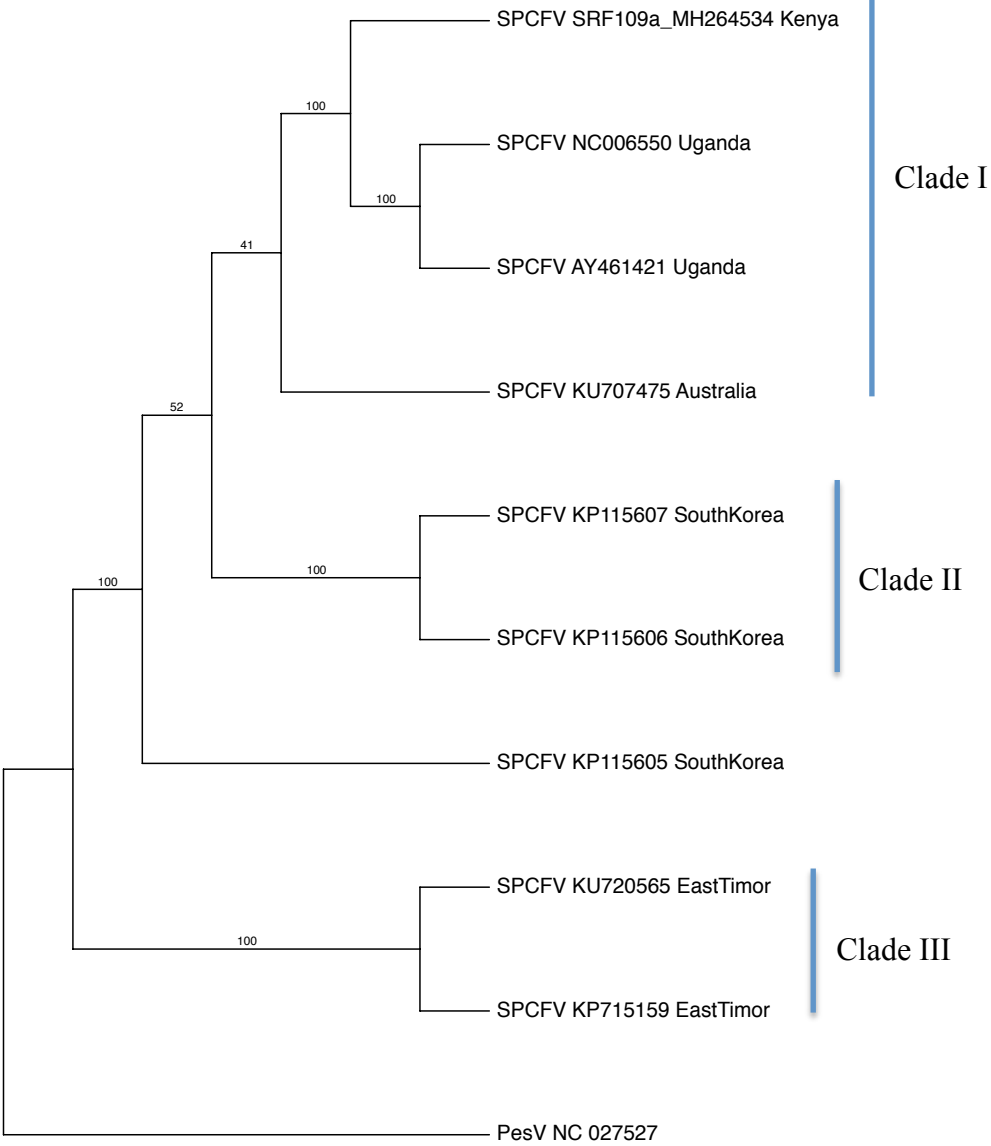

Supplement: Supplemental Information 6 — The nodes across each branch are labeled with bootstrap values. Tip labels contain information of: virus name, GenBank accession number and/or field identification and country where sampling was conducted. [file peerj-06-5254-s006.pdf]

SVDQuartets bootstrap consensus

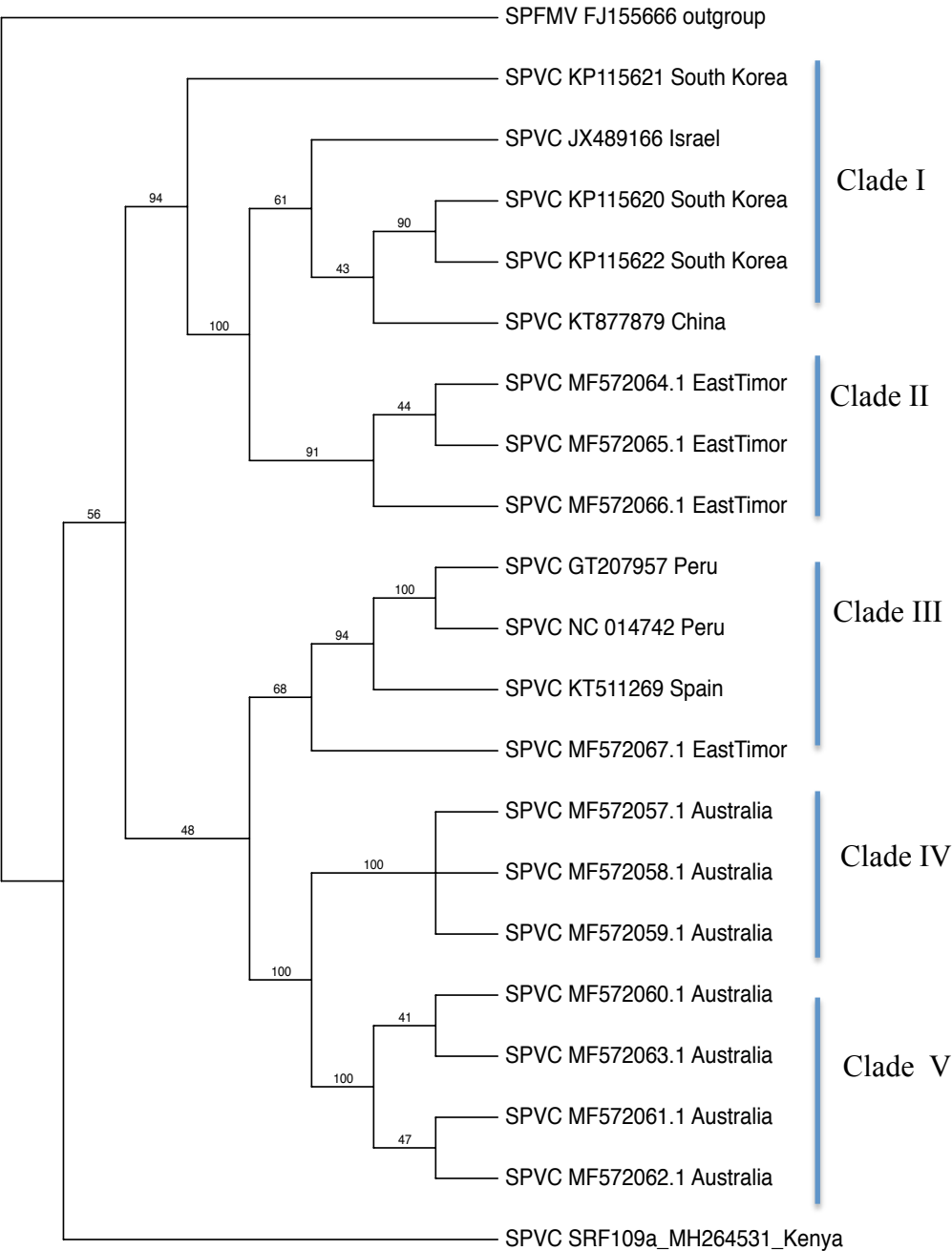

Supplement: Supplemental Information 7 [file peerj-06-5254-s007.pdf]

SVDQuartets bootstrap consensus

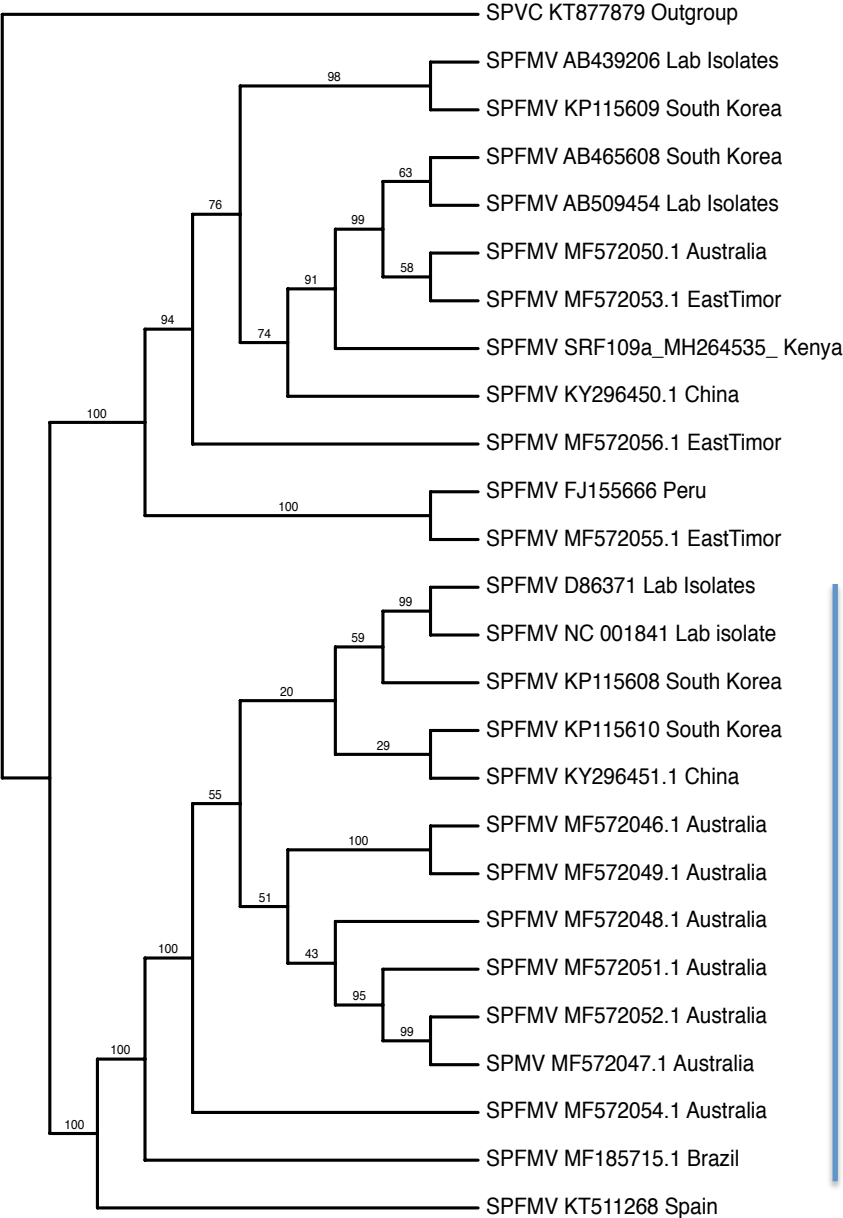

Clade I

Clade II

Supplement: Supplemental Information 8 — The nodes across each branch are labeled with bootstrap values. Tip labels contain information of: virus name, GenBank accession number and/or field identification and country where sampling was conducted. [file peerj-06-5254-s008.pdf]
